# Concurrent insulinoma and pancreatic adenocarcinoma: report of a rare case and review of the literature

**DOI:** 10.1186/1477-7819-9-7

**Published:** 2011-01-25

**Authors:** Panagiotis G Athanasopoulos, George Polymeneas, Dionysios Dellaportas, George Mastorakos, Evi Kairi, Dionysios Voros

**Affiliations:** 1 Department of Surgery, University of Athens, Aretaieion University Hospital, 76 Vas. Sofias Ave., 11528, Athens, Greece; 2 Unit of Endocrinology, University of Athens, Aretaieion University Hospital, 76 Vas. Sofias Ave., 11528, Athens, Greece; 3 Department of Pathology , University of Athens, Aretaieion University Hospital, 76 Vas. Sofias Ave., 11528, Athens, Greece

## Abstract

Pancreatic adenocarcinoma is the 5th leading cause of cancer-related death in Western countries and insulinomas are rare endocrine neoplasms of the pancreas. The concurrent appearance of pancreatic adenocarcinoma and insulinoma is very rare and to the best of our knowledge has never been reported again. Herein, we present such an occurrence in a 74-year-old man. Resection of a mass in the uncinate process of the pancreas revealed pancreatic adenocarcinoma with severe desmoplastic reaction. Two years later, due to symptomatology persistence the patient was re-examined and a new 2cm mass in the uncinate process was found leading to surgery, which demonstrated a 2cm endocrine islet-cell tumor. Establishing a diagnosis in patients with insulinoma is difficult and the imaging studies still have low sensitivity and specificity except for intra-operative ultrasonography, which is the most accurate method detecting 90% of these lesions.

## Background

Pancreatic endocrine neoplasms are rare tumours with a reported incidence of four cases per million patients per year [1]. Of these tumours, insulinomas are the most common and typically are sporadic and solitary masses affecting individuals 30-60 years old, with equal distribution among genders [2]. On the other hand, pancreatic adenocarcinoma is the 5th leading cause of cancer death in Western countries, and the second cause of cancer death among gastrointestinal tumors [3]. An unusual occurrence of concomitant pancreatic adenocarcinoma and insulinoma in a 74-year-old man is presented herein.

## Case Presentation

A 74-year-old man was admitted to our hospital, suffering for the last 2 years from hypoglycaemic attacks. Laboratory tests showed fasting glucose level below 50mg/dl and symptoms of hypoglycaemia such as tachycardia, sweating, confusion and light-headedness. The correction of the above when glucose was administered was significant, so the Whipple's triad was present. Plasma-insulin level, measured through the extended 72-hours fasting test was found 12mIU/ml and C-peptide level was also elevated, 4.3ng/ml. Tumour markers were within normal range apart from Ca 19-9 which was 5IU/ml.

**Figure 1:**
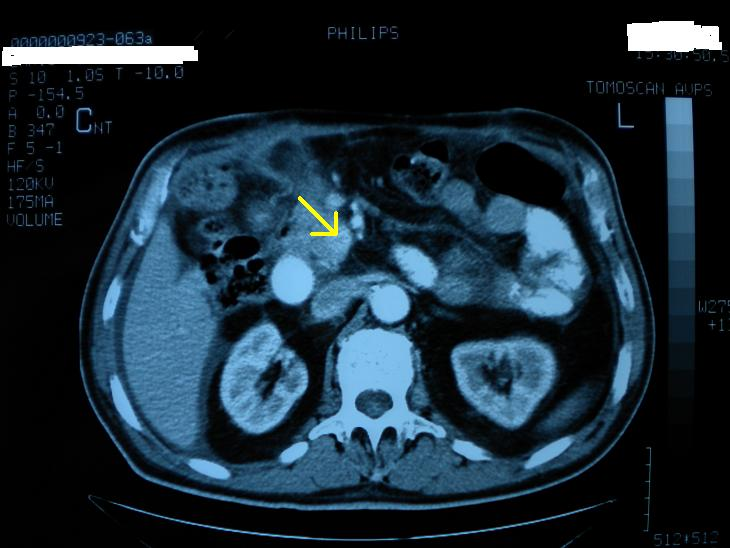
Contrast enhanced CT scan depicting the 1.5 cm solid mass in the uncinate process of the pancreas (arrow).

**Figure 2:**
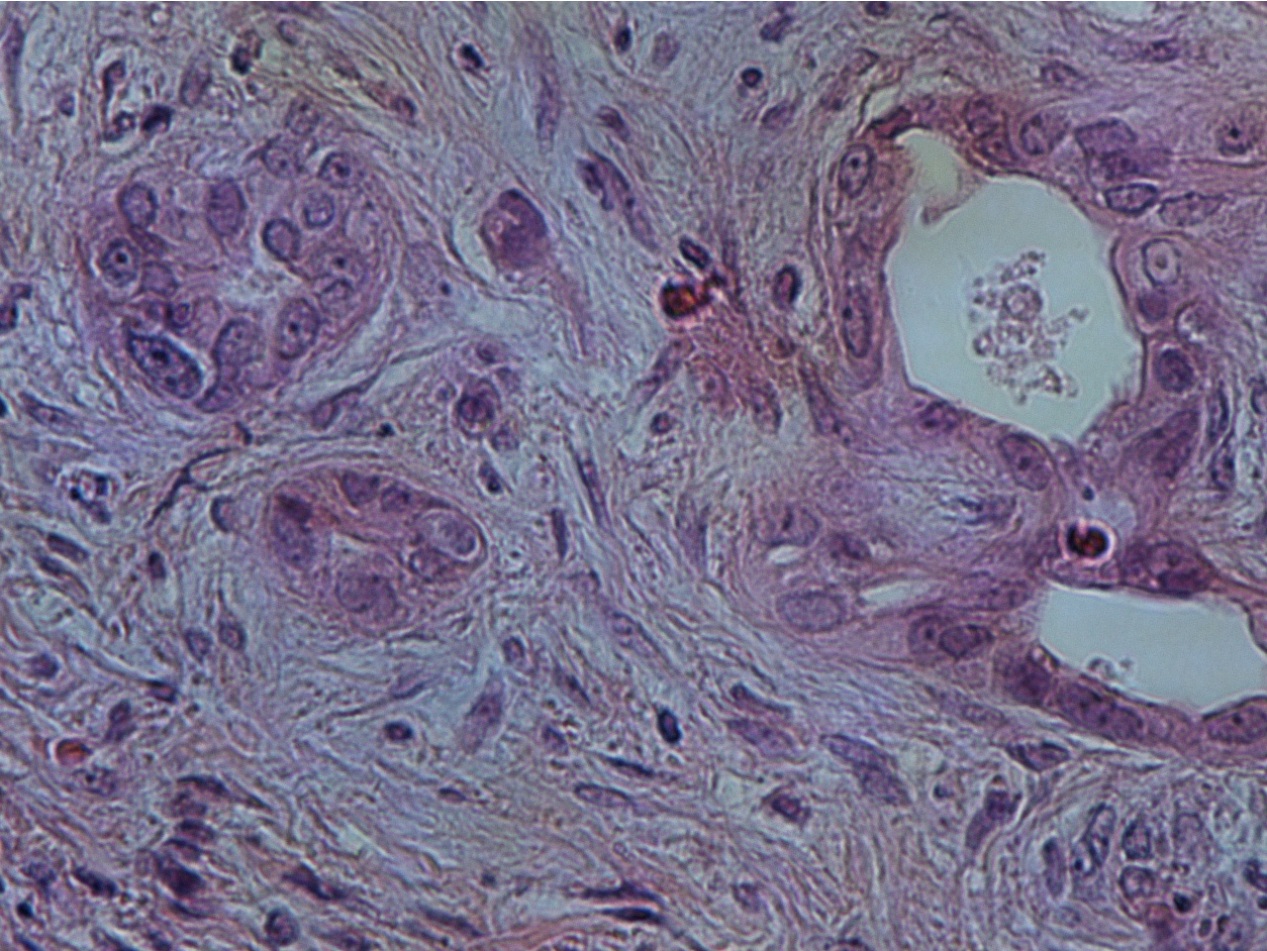
Microscopic focus of pancreatic adenocarcinoma with desmoplastic reaction (Hematoxylin-Eosin × 400).

Abdominal ultrasonography (US) did not reveal any lesion, but a contrast enhanced CT scan demonstrated a 1.5cm solid mass in the uncinate process of the pancreas (Figure 1). The patient underwent surgical exploration and pancreas palpation indeed revealed a small solid mass in the uncinate process, which was resected with a small amount of normal tissue surrounding the mass. No frozen section biopsy was done since we believed that the mass represented the insulinoma. However, the histopathological study featured a pancreatic adenocarcinoma of about 1mm in maximum diameter with intense desmoplastic reaction around the lesion (Figure 2). We estimated that the performed resection was enough treatment for such a small carcinoma. The patient's postoperative course was uneventful and he was discharged on the seventh postoperative day with a regular follow-up as the only recommendation.

**Figure 3:**
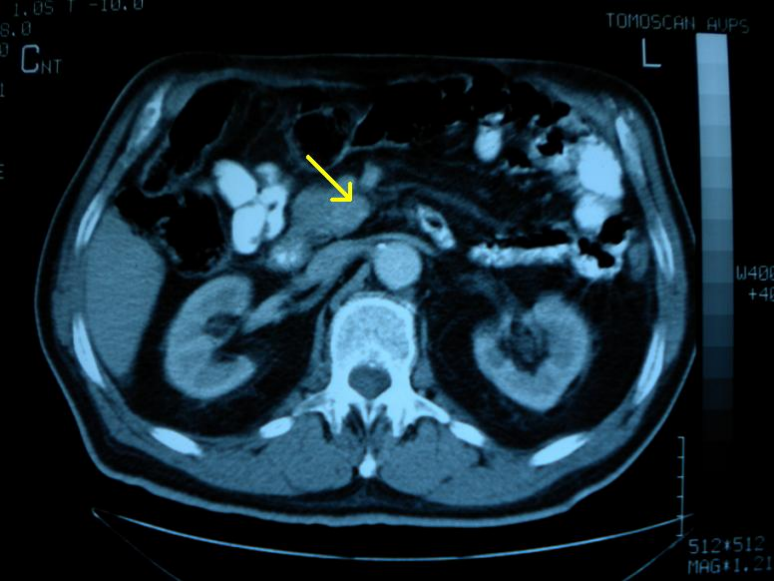
Contrast enhanced CT scan, after the first operation, demonstrating a 2 cm mass in the uncinate pancreatic process, with characteristics of an endocrine neoplasm (arrow).

**Figure 4:**
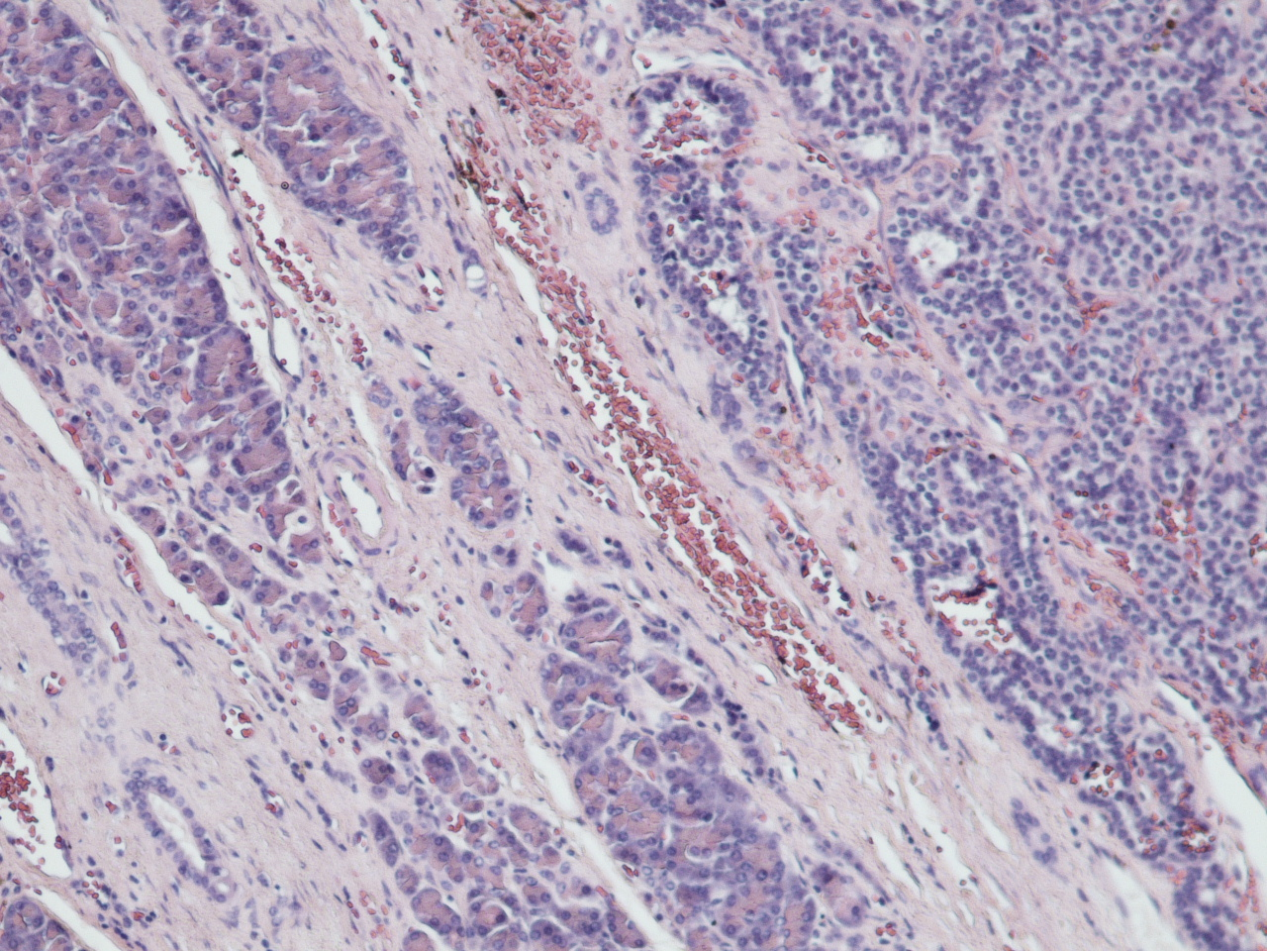
Pancreatic endocrine tumour ("insulinoma") adjacent to the pancreatic parenchyma (Hematoxylin-Eosin × 100).

**Figure 5:**
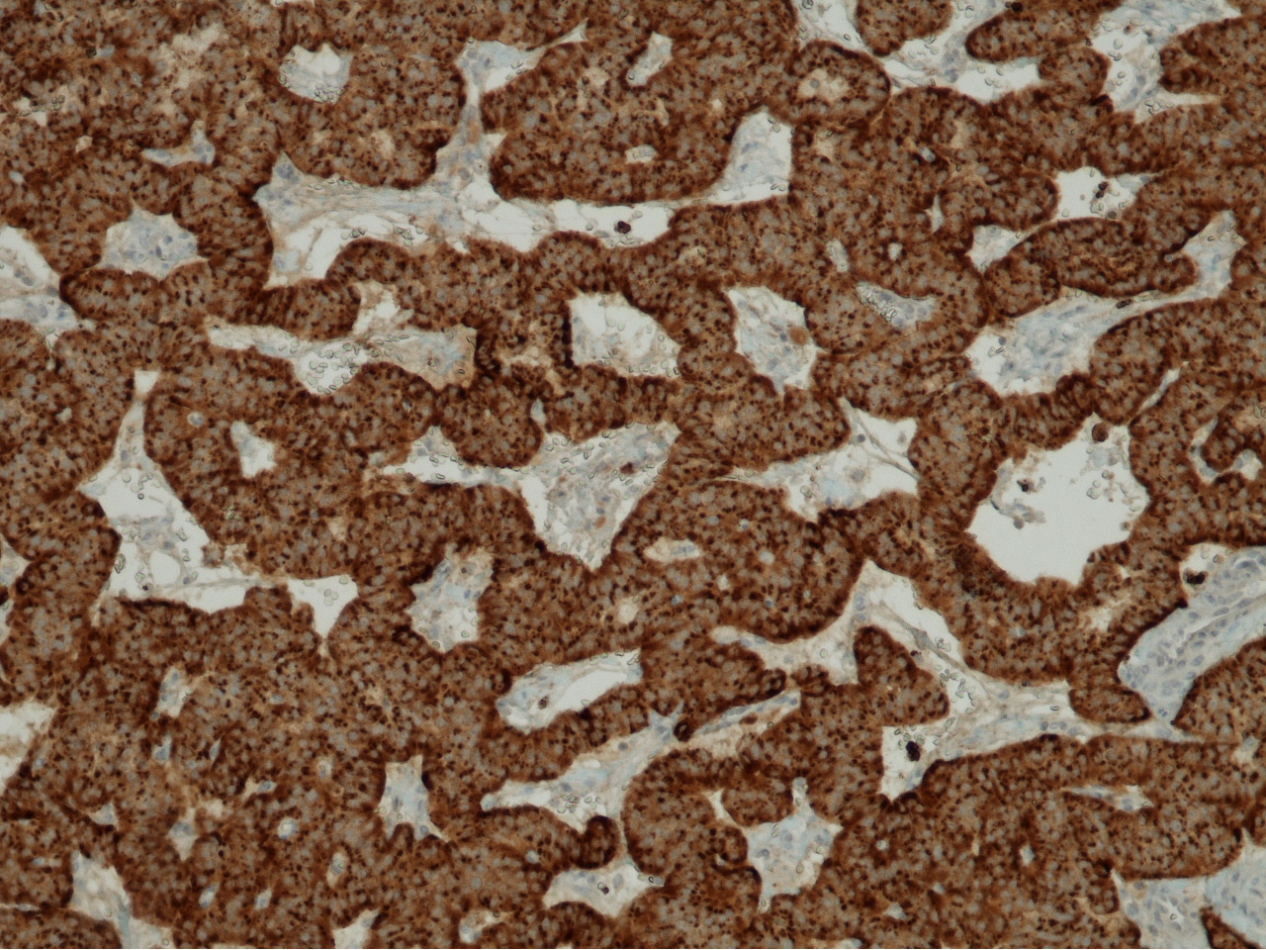
Pancreatic endocrine tumour, intensely immunostained for insulin (x 100).

Serial evaluation of Ca 19-9, at 3-month intervals, during the following two years, was found within normal range. Due to persistence of hypoglycaemic symptoms, successive abdominal CT scans completed the follow-up [4] but they were inconclusive because of the increased postoperative inflammatory reaction in the region of the resection. Thus, hypoglycaemic symptoms were classified as idiopathic and were significantly improved with appropriate dietary modifications. After two years, better CT image resolution presumably due to regression of the inflammatory phenomena, rendered the insulinoma mass detectable again, having at that time increased its size from 1.5cm to 2cm (Figure 3). Another operation was decided and pancreaticoduodenectomy was performed. The histopathological examination revealed a 2cm endocrine islet-cell tumour (Figure 4, 5) and the patient was discharged on the tenth postoperative day.

## Discussion

The main clinical symptom in insulinoma patients is the inability to suppress insulin secretion in the presence of hypoglycaemia, resulting in neuroglycopenia and adrenergic manifestations like headache, confusion, visual troubles, shivering, irritability and palpitations [5]. However, establishing a diagnosis in patients with insulinoma is difficult and the imaging studies still have low sensitivity and specificity. The sensitivity of abdominal US is 50% whereas in contrast enhanced CT scan is 24%, in MRI scan is 40% and in scintigraphy using octreotide approaches 60%. Endoscopic US is the most accurate non-interventional imaging modality detecting 77% of the pancreatic insulinomas [6,7]. However, intraoperative US is even more accurate detecting 90% of these lesions, which are usually smaller than 2cm in maximum diameter [8].

In our case, palpation of the lesion in the uncinate process during the first operation thought to be the mass, which had been demonstrated on the preoperative abdominal CT. Unfortunately, we missed the 1.5cm insulinoma and the resected area revealed a 1mm pancreatic adenocarcinoma with severe desmoplastic reaction in the surrounding tissue, which when palpated obviously misled us to believe that it corresponded to the insulinoma. The patient's symptoms pertained and serial CT scans revealed the 2cm insulinoma which was successfully treated with a Whipple's procedure.

Mixed pancreatic tumours, either collision or composite ones, from endocrine and exocrine cells have been reported in the literature [9], as well as the coexistence of insulinoma and gastrointestinal stromal tumours especially in patients with neurofibromatosis type I [10]. However, the concurrence of pancreatic adenocarcinoma and insulinoma has never been reported before.

## Conclusions

The coexistence of pancreatic adenocarcinoma and insulinoma is very rare and has never been reported before. Clinical symptoms should be evaluated carefully and since imaging modalities have low sensitivity and specificity in detecting small endocrine neoplasms, sequential imaging studies and intraoperative US can prove very helpful.

## Consent

Written informed consent was obtained from the patient for publication of this case report and any accompanying images. A copy of the written consent is available for review by the Editor-in-Chief of this journal.

## Competing interests

The authors declare that they have no competing interests.

## Authors' contributions

PA participated in the surgical procedure, conceived and designed the study, and wrote the manuscript. GP analysed the data and drafted critically the manuscript. DD participated in the surgical procedure, acquired the data and helped in writing the manuscript. GM helped in the acquisition and interpretation of data, and he drafted the manuscript. EK performed the appropriate histological analysis of the surgical specimens and provided histological sections as figures for the manuscript. DV carried out the surgical procedure, participated in designing the study and finally revised the manuscript for submission. All authors read and approved the final manuscript.

## References

[B1] Service FJ McMahon MM O'Brien PC Ballard DJ Functioning insulinoma- incidence, recurrence and long-survival of patients: a 60-year study Mayo Clin Proc 1991 66 711 719 1677058 10.1016/s0025-6196(12)62083-7

[B2] Kuzin NM Egorov AV Kondrashin SA Lotov AN Kuznetzov NS Majorova JB Preoperative and intraoperative topographic diagnosis of insulinomas World J Surg 1998 22 593 597 10.1007/s002689900440 9597934

[B3] Jemal A Murray T Ward E Samuels A Tiwari RC Ghafoor A Feuer EJ Thun MJ Cancer statistics, 2005 CA Cancer J Clin 2005 55 10 30 10.3322/canjclin.55.1.10 15661684

[B4] Zervos EE Rosemurgy AS Al-Saif O Durkin AJ Surgical management of early-stage pancreatic cancer Cancer Control 2004 11 23 31 14749620 10.1177/107327480401100104

[B5] Grant CS Gastrointestinal endocrine tumours. Insulinoma Baillieres Clin Gastroenterol 1996 10 645 671 10.1016/S0950-3528(96)90017-2 9113316

[B6] Grant CS Surgical aspects of hyperinsulinemic hypoglycemia Endocrinol Metab Clin North Am 1999 28 533 554 10.1016/S0889-8529(05)70087-6 10500930

[B7] Gibril F Reynolds JC Doppman JL Chen CC Venzon DJ Termanini B Weber HC Stewart CA Jensen RT Somatostatin receptor scintigraphy: its sensitivity compared with that of other imaging methods in detecting primary and metastatic gastrinomas. A prospective study Ann Intern Med 1996 125 26 34 8644985 10.7326/0003-4819-125-1-199607010-00005

[B8] Hashimoto LA Walsh RM Preoperative localization of insulinomas is not necessary J Am Coll Surg 1999 189 368 373 10.1016/S1072-7515(99)00163-5 10509462

[B9] Capella C La Rosa S Uccella S Billo P Cornaggia M Mixed endocrine-exocrine tumors of the gastrointestinal tract Semin Diagn Pathol 2000 17 91 103 10839609

[B10] Teramoto S Ota T Maniwa A Matsui T Itaya N Aoyagi K Kusanagi H Narita M Two von Recklinghausen's disease cases with pheochromocytomas and gastrointestinal stromal tumors (GIST) in combination Int J Urol 2007 14 73 74 10.1111/j.1442-2042.2006.01601.x 17199864

